# Molecular dynamics simulation on the reaction of nano-aluminum with water: size and passivation effects

**DOI:** 10.1039/c9ra08484c

**Published:** 2019-12-17

**Authors:** Rui-Kang Dong, Zheng Mei, Feng-Qi Zhao, Si-Yu Xu, Xue-Hai Ju

**Affiliations:** School of Chemical Engineering, Nanjing University of Science and Technology Nanjing 210094 P. R. China xhju@njust.edu.cn; Laboratory of Science and Technology on Combustion and Explosion, Xi'an Modern Chemistry Research Institute Xi'an 710065 P. R. China

## Abstract

The reaction of aluminum and water is widely used in the field of propulsion and hydrogen production, but its reaction characteristics at the nanometer scale have not been fully studied. In this paper, the effect of particle size and surface passivation of aluminum particle on the reaction mechanism was studied by using reactive molecular dynamics (RMD) simulation. The reduction of aluminum particle size can accelerate the reaction rate in the medium term (20–80 ps) due to the increase of activity, but it also produces an agglomeration effect as the temperature increases. The presence of surface passivation reduces the proportion of active aluminum and the yield of hydrogen decreases by 30% and 33%, respectively, as the particle size decreases from 2.5 nm to 1.6 nm. The addition of AlH_3_ can overcome these drawbacks when some aluminum powders are replaced by AlH_3_. The hydrogen yield is increased by the reaction 2AlH_3_ + 3H_2_O → Al_2_O_3_ + 6H_2_. In the reaction of surface passivated Al (1.6 nm in diameter) and H_2_O, when the proportion of AlH_3_ reaches 25%, the energy release and hydrogen yield increase from 59.47 kJ mol^−1^ and 0.0042 mol g^−1^ to 142.56 kJ mol^−1^ and 0.0076 mol g^−1^, respectively. This performance even approximates the reaction of pure aluminum with water: 180.67 kJ mol^−1^ and 0.0087 mol g^−1^. In addition, the surface passivation affects the reaction mechanism. Before the passivation layer melts, the reaction 4Al + Al_2_O_3_ → 3Al_2_O occurs inside the nanoparticles.

## Abstract 2

The temperature distribution, number of Al–O bonds and hydrogen yield evolution with time.

## Graphical abstract

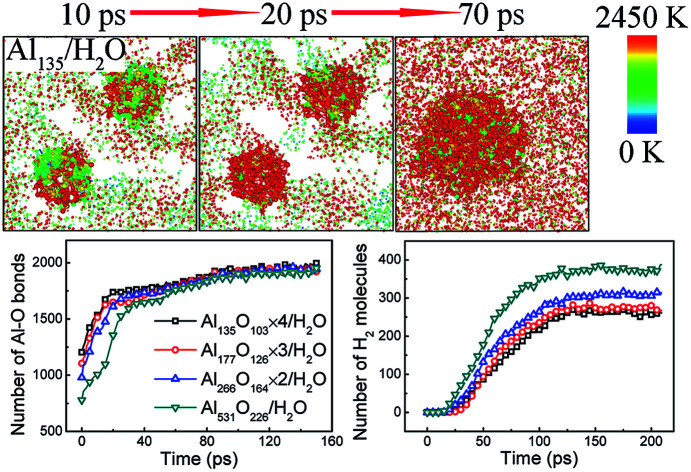

## 1 Introduction

The aluminum–water reaction has attracted the attention of many researchers in recent decades due to its high energy and environmentally friendly products.^1–7^ The aluminum–water mixture is made by mixing aluminum powder and ice, so researchers call it ALICE. ALICE is considered a new high-energy material that can be widely used in hydrogen production, underwater propulsion and space propulsion.^8–11^ Unlike the conventional methods of producing hydrogen from petroleum, which release many harmful by-products such as carbon dioxide, hydrogen production from aluminum and water is not only simple but also pollution-free.^12–15^ Moreover, the convenience of obtaining water enables hydrogen production anytime and anywhere. The low density and stability of aluminum greatly save transportation costs and improve the security of storage.

The aluminum–water reaction produces hydrogen and releases a lot of heat. Theoretically, aluminum–water mixture could be prepared *in situ* on any water-bearing planet. Therefore, researchers are considering it as a new energy source for propellant applications. In 2009, NASA's ALICE propellant sounding rocket was successfully launched, which marked that the development of such propellants had achieved a stage achievement, and proved the application of aluminum–water mixture in space propulsion.^16^ The wide development prospect of aluminum–water reaction makes it become a research upsurge.

The particle sizes of aluminum used in the early study were mostly micron or larger, which lead to the aluminum–water combustion need to be carried out at a high temperature and difficulty in ignition. In recent years, with the emergence of nanotechnology, micron aluminum powder is replaced by nano-aluminum powder to improve the combustion performance of the aluminum–water mixture. Ivanov *et al.* first investigated the combustion behavior of nano-aluminum/water mixture. They mixed the nano-aluminum powder more evenly with water by adding polyacrylamide with a mass fraction of 3%. The results showed that the reaction could continue in the temperature range of 323–348 K. In addition, the combustion rate and hydrogen production increase as the temperature increases.^17^ After that, Risha *et al.* tried to study the combustion characteristics of aluminum–water mixture without adding any gels. The aluminum–water mixture was added into an 8 mm diameter quartz tube and ignited in a combustion chamber filled with argon. The results showed that the combustion rate decreases with the increase of mixture equivalence ratio. The main reason is that equivalence ratio affects the flame temperature and thus changes the combustion state.^18^ Risha also studied the effect of aluminum particle size on combustion efficiency of aluminum–water mixture. As the particle size decreases from 130 nm to 38 nm, the combustion efficiency increases from 27% to 95%.^19^ Sundaram *et al.* studied the influence of aluminum particle size and pressure on the combustion rate of aluminum–water mixture by means of theoretical analysis and experiment. The particle size of aluminum powder used in the experiment was 38–130 nm, and the pressure range considered was 1–10 MPa. The results showed that the combustion rate is proportional to the pressure and inversely proportional to the particle size.^20^

However, the application of nano-sized aluminum particle also brings some problems. Aluminum particles usually undergo surface passivation due to their high activity. Although the passivation layer can improve the stability of aluminum particles, it reduces their energy density. With the decrease of particles size, the specific surface area of nano-aluminum increases and the presence of passivation layer bring significant influence: the proportion of active aluminum decreases obviously.^18^ Entrainment and agglomeration of nanoparticles also affect the combustion of nano-aluminum/water mixture. Sundaram *et al.* investigated this project using 80 nanometer aluminum–water mixture. When considering the entrainment action of aluminum particles, the flame thickness is increased by 10 times. As the entrainment parameter increases from 0 to 1.0, the pressure index increases from 0 to 0.5, and the combustion rate decreases correspondingly. It is considered that the agglomeration of aluminum particles with diameter of 3–5 μm leads to the combustion from kinetic control to diffusion control.^9^

To solve these problems, researchers are actively looking for high-energy additives. Hydrogen peroxide is a highly reactive liquid with strong oxidation properties. Its addition can improve the combustion performance of aluminum–water mixture. However, high concentrations (>32%) of hydrogen peroxide may cause the mixture to burn with deflagration, break down easily in storage, or be unstable in transport.^21,22^ Moreover, the addition of hydrogen peroxide reduces the hydrogen yield in the reaction of aluminum with water, and the gas yield has a great influence on the specific impulse of propellant.

AlH_3_ as a reducing agent has the advantages of high combustion heat, high specific impulse, and non-toxicity. It can be used as an excellent propellant additive.^23–28^ Introducing AlH_3_ into aluminum–water mixture instead of some aluminum particles can effectively reduce the influence of aluminum surface passivation. Connell *et al.* combined experiment and simulation to study the combustion phenomenon of nano-ALICE propellant when some aluminum powders were replaced by AlH_3_. Small quantities of AlH_3_ added into mixture can increase the specific impulse by 10%, although the flame temperature is reduced by 5%. When the mass fraction of AlH_3_ increases to 25%, the specific impulse of ALICE propellant increases from 210 s to nearly 230 s. Meanwhile, the addition of AlH_3_ increases the hydrogen yield and reduces the production of alumina.^29,30^

At present, most studies on Al/AlH_3_/H_2_O mixture were carried out through experiments. A few theoretical researches were devoted to thermo-chemistry equilibrium calculation or the establishment of multi-zone theoretical combustion framework.^26,27^ The complete reaction process has not been elucidated through simulation. In fact, understanding the reaction mechanism can be a good guide to improve the performance of Al/AlH_3_/H_2_O propellants. In this paper, we will demonstrate the influence of nano-aluminum particle size and surface passivation on the reaction of aluminum and water, and explore the combustion performance of Al/H_2_O mixture after the addition of AlH_3_.

Reactive molecular dynamics (RMD) is a suitable method for simulating chemical reaction process. The RMD can calculate the instantaneous position and velocity of the atoms through ReaxFF, a force field developed by van Duin.^31–35^ Russo *et al.* used the ReaxFF to simulate the dissociation process of H_2_O molecules on the surface of Al_100_ cluster at 1650 K temperature.^36^ They focused on the dissociation behavior of O–H bonds in systems with different concentrations of H_2_O, and found that higher concentrations of H_2_O are favorable for the dissociation. The reaction is that the dissociation of H_2_O molecules adsorbed on the surface of Al_100_ requires a large amount of activation energy (*E*_a_), but it is easier to dissociate with the assistance of adjacent, non-adsorbed H_2_O molecules.

Li *et al.* parameterized a new ReaxFF suitable for atomic systems containing C/H/O/N/Al to study the thermal decomposition of RDX with AlH_3_.^37^ We used this parameterized ReaxFF to study the combustion behavior of Al/AlH_3_/H_2_O mixtures. Four aluminum–water systems with different aluminum particle sizes were established to demonstrate the effect of particle sizes on reaction rates. Then the corresponding aluminum particles were coated with a passivated layer of uniform thickness, and the effect of the surface passivation on the reaction with the change of particle size was studied. Finally, a part of surface passivated aluminum particles were replaced by AlH_3_ particles to study the effect on the reaction performance.

## 2 Modeling and calculation methods

**Fig. 1:**
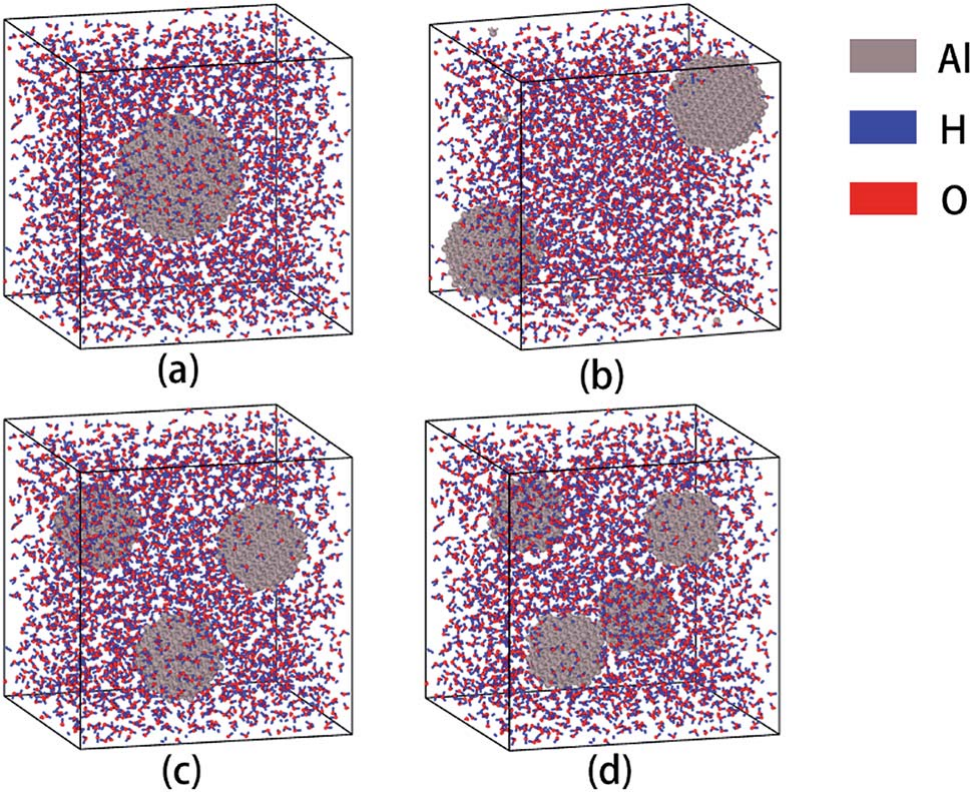
Supercells containing Al–H_2_O mixtures of different aluminum particle sizes. The diameters of Al particles are 2.50 (a), 2.04 (b), 1.72 (c) and 1.60 (d) nm, respectively.

A periodic supercell filled with H_2_O molecules (5.5 nm × 5.5 nm × 5.5 nm) was constructed through Amorphous Cell Modules of Materials Studio Software, wherein the density of H_2_O is 0.5 g cm^−3^. The concentration of H_2_O depends on the reaction environment. The reaction zone is located at the combustion front plane, which represents a solid–liquid–gas state, and contains solid aluminum particles, water liquid and vapor. In this state, the density is less than the condensed fuel, but greater than the vapor. Then part of the H_2_O molecules in the center of the cell were removed and replaced with an aluminum particle. At this point, the modeling of an Al/H_2_O mixed supercell was completed. Al particles were established in Materials Studio through Build → Build nanostructure → Nanocluster, and then the structures were optimized to minimize the energy. Next, in order to investigate the effect of particle size on the reaction, we established Al/H_2_O mixed supercells containing aluminum particles with different particle size using the same method. In order to control the variables, we reduced the particle size while increasing the number of aluminum particles with more number of particles, so that the number of aluminum atoms in each cell was basically the same. The completed models are shown in Fig. 1, and the detailed parameters are listed in Table 1. These models are used to study the effect of particle size on aluminum–water reaction.

**Table 1:** Detailed parameters of aluminum-water composite models^a^

Al diameter (in nm)	Cell ingredients	Number of atoms
2.50	2585 H_2_O + Al_531_	8286
2.04	2565 H_2_O + 2×Al_266_	8227
1.72	2563 H_2_O + 3×Al_177_	8220
1.60	2538 H_2_O + 4×Al_135_	8154

a The model size is 5.5 × 5.5 × 5.5 nm.

**Fig. 2:**
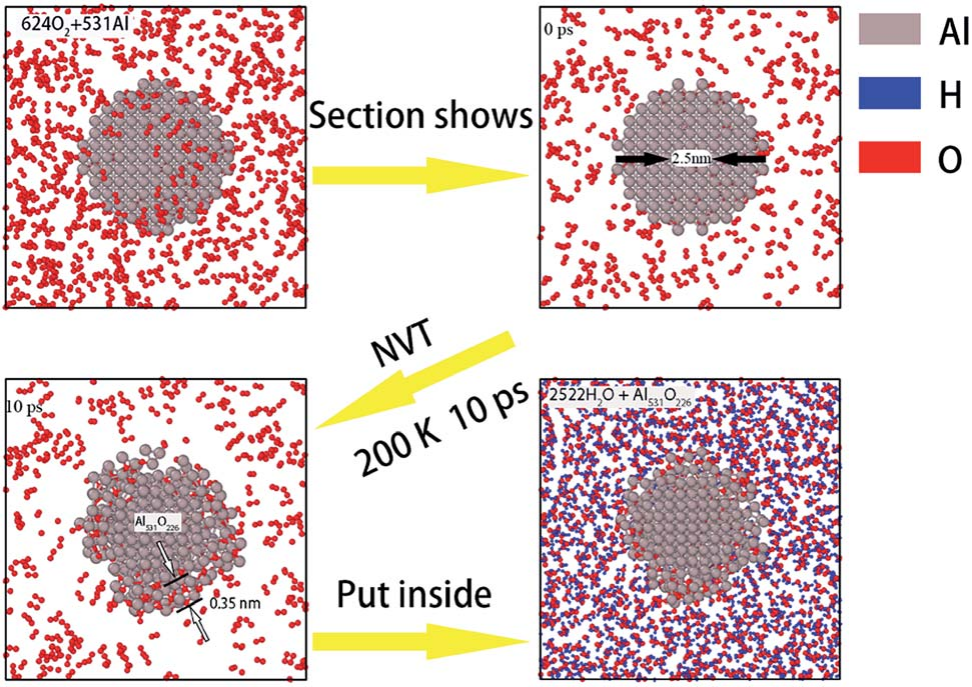
Formation of surface passivation of aluminum particles.

Next, in order to study the effect of surface passivation on aluminum–water reaction, we passivated the surfaces of aluminum particles through surface oxidizing. To ensure the uniform thickness of all passivated layers, pure aluminum particles were placed separately in supercells filled with oxygen and heated uniformly for 10 ps at 200 K. The formation process of the passivated layer is shown in Fig. 2.

Fig. 2 shows the formation process of passivated layer on the surface of the aluminum particle with a diameter of 2.50 nm. A cell filled with O_2_ molecules was established by the Amorphous Cells modules in Materials Studio Software. After that, part of the O_2_ molecules were hollowed out from the center of the cell to obtain a spherical cavity and an aluminum particle of 2.5 nm in diameter was put into it. The passivated process was performed using the LAMMPS package by the ReaxFF force field. The system was heated by 10 ps at a temperature of 200 K under NVT canonical ensemble to simulate the surface oxidation process. After 10 ps, O atoms adsorb on the surface of Al particle, bond with Al atoms and form a 0.35 nm thick oxide layer on the surface. Finally, this surface passivated Al particle was placed into the center of the supercell filled with H_2_O molecules established above (with a density of 0.5 g cm^−3^ and size of 5.5 nm × 5.5 nm × 5.5 nm) in the same manner. The surface of Al particles became rugged after passivated and thus occupied more space, leading a slight decrease in the number of H_2_O molecules in the system (from 2585 to 2522). Since the number of H_2_O molecules is much larger than that of Al atoms, the variation of oxygen atoms has little effect on the simulation results.

**Fig. 3:**
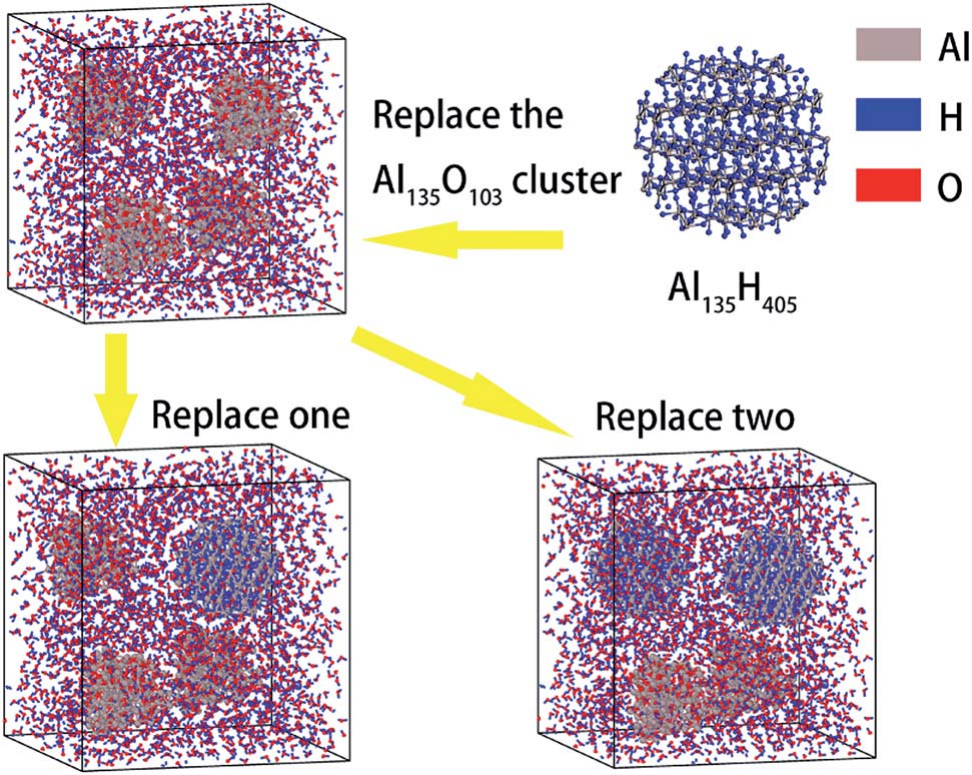
Replacement of surface passivated aluminum particles by AlH_3_ particles.

Next, AlH_3_ particles were used to replace part of the surface passivated aluminum particles to study the effect of AlH_3_ on the reaction performance. The construction process of AlH_3_ particles is consistent with that of Al particles. The modeling details are shown in Fig. 3. In the supercells containing 1.60 nm aluminum particles, the molar ratio of AlH_3_ reached 0, 25% and 50%, respectively.

**Fig. 4:**
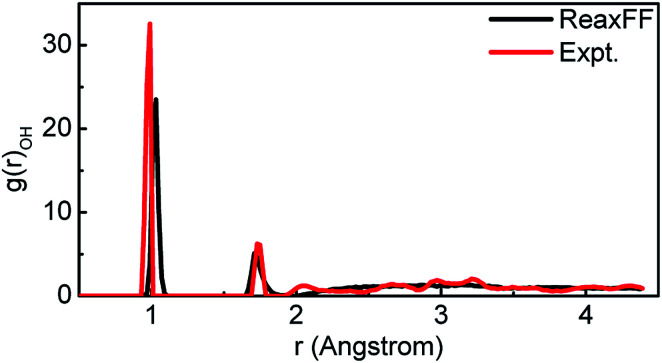
The comparison of the radial distribution function (RDF) of O–H in liquid water calculated by ReaxFF and experiment value.

For comparison, all the supercells above are the same size. Reactive molecular dynamics (RMD) simulations of these supercells were performed in a uniform environment by utilizing parametric ReaxFF. The suitability of this parameterized ReaxFF is supported by the information in Table 2, 3 and Fig. 4. The bond length and bond angle of H_2_O molecule calculated from ReaxFF were compared to the DFT values and experimental results in Table 2.^38^ The lattice parameters of H_2_O supercell simulated by ReaxFF were compared to both the calculated values of Generalized Gradient Approximation (GGA) and experiment in Table 3. Finally, we compared the radial distribution function (RDF) of H_2_O molecules optimized by ReaxFF with the experimental values in Fig. 4. The first peak of *r* represents the length of the O–H bonds of H_2_O molecules. The length of O–H in H_2_O optimized by ReaxFF is 1.01 Å, while the experimental value is 0.97 Å. The second peak represents the length of the intermolecular hydrogen bond. The optimized value from ReaxFF is 1.71 Å, while the experimental value is 1.75 Å. The error between the simulation value and the experimental value is within 5%.^38^

**Table 2:** Bond length and bond angle of H_2_O calculated from COMPASS and parameterized ReaxFF

Methods	*r*_O–H_ a	RE%	∠H–O–H^b^	RE%^c^
Expt^d^	0.966	—	102.80	—
COMPASS	0.949	−1.76	104.49	1.64
ReaxFF	1.011	4.66	100.88	−1.87

a *r*_O–H_ is the O–H length in Angstrom.

b ∠H–O–H is the bond angle in degree.

c Relative error.

d Experimental values from ref. 38.

**Table 3:** Lattice parameters of H_2_O calculated from GGA and ReaxFF^a^

Methods	*a*	*b*	*c*	*α*	*β*	*γ*
Expt^b^	7.800	4.500	5.560	90.00	90.00	90.00
GGA/PBE	7.660(−1.79)	4.370(−2.89)	5.981(7.57)	90.22(0.24)	90.04(0.04)	89.76(−0.27)
GGA/RPBE	7.858(0.74)	4.585(1.89)	6.032(8.49)	90.00(0.00)	90.00(0.00)	90.01(0.01)
GGA/PW91	7.318(−6.18)	4.012(−10.84)	5.902(6.15)	89.81(−0.21)	90.11(0.12)	86.56(−3.82)
ReaxFF	7.144(−8.41)	4.122(−8.40)	5.092(−8.42)	90.00(0.00)	90.00(0.00)	90.00(0.00)

a The values in parentheses are the relative errors (%).

b Experimental values are from ref. 39.

The application of canonical ensemble (NVT) was to obtain the canonical distribution of kinetic energy and potential energy of isothermal and isovolumetric systems. For optimization, the temperature of each system was maintained at 1 K for 10 ps under NVT conditions to obtain a stable initial structure. It was then heated at 35 K ps^−1^ to 3500 K and kept constant for 100 ps to make sure the reaction is complete.

All the RMD simulations were performed using LAMMPS program package with a neighbor force cutoff of 0.6 nm and a time step of 0.25 fs. Meanwhile, the Nose–Hoover thermostat was employed in the simulations. In order to distinguish the chemical species, one must judge the bonding status between neighbor atoms. In this work, the judgement is based on the distance between atoms. A bond is deemed to form only when the distance is smaller than the product of standard single-bond length and a scale factor. The scale factor is set to 1.2 for the tolerance of vibrations.

The thermodynamic state is output once every 4000 steps of calculation (the time interval is 1 ps as the time step is 0.25 fs). Therefore, the simulation covers the reaction process of 200 ps, excluding the relaxation time of 10 ps.

## 3 Results and discussion

### 3.1 Reaction rate of pure Al particle

**Fig. 5:**
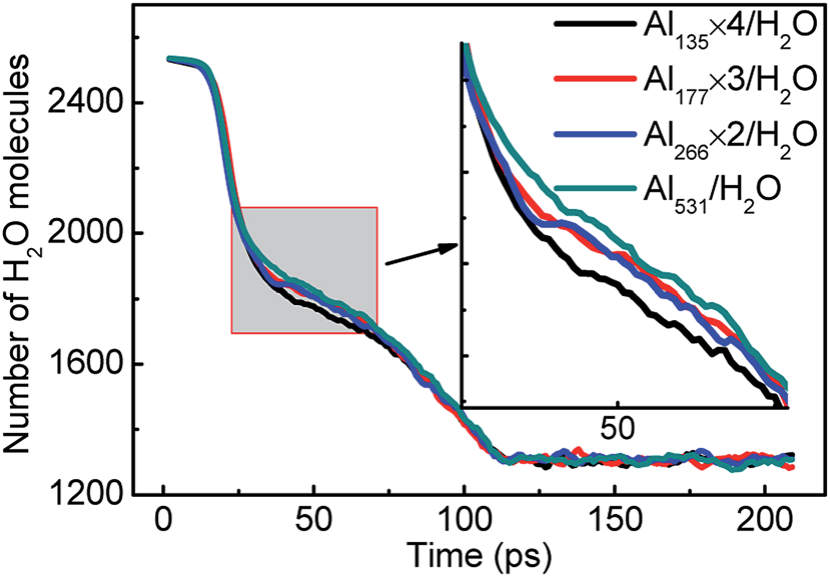
Consumption of H_2_O molecules in the reactions of aluminum and water with different aluminum particle sizes.

The reaction rate of the system can be expressed by the consumption rate of H_2_O molecules. Fig. 5 compares the consumption rate of H_2_O molecules by aluminum with different particle sizes. The number of H_2_O molecules is the same in the initial and final stages of each system, which indicates that the reaction equilibrium is not affected by particle size. But over a period of 20–70 ps, the difference in the reaction rates can be seen quite clearly. The consumption rate of H_2_O increases with the decrease of aluminum particle size. The increase of the specific surface area of aluminum particles is one of the reasons.

**Fig. 6:**
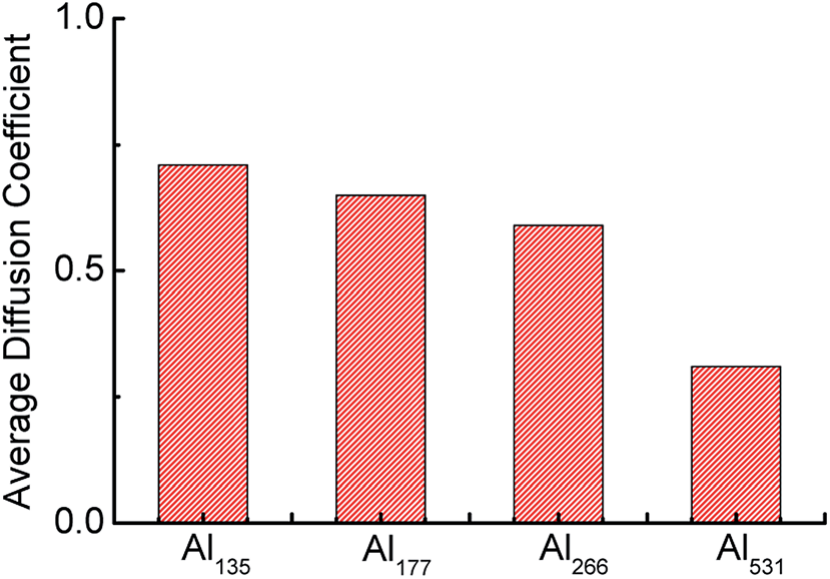
Average diffusion coefficients of aluminum with different particle sizes.

In addition, as an indicator of atomic motion, the diffusion coefficient can reflect the activity of atoms in chemical reactions. The average diffusion coefficients throughout the whole reaction of aluminum with different particle sizes are shown in Fig. 6. The average diffusion coefficient of Al_135_ is the largest and that of Al_531_ is the smallest, which indicates that as the particle size of the aluminum particle decreases, the aluminum atoms therein are more active and easily react with H_2_O. This is the second reason that the consumption rate of H_2_O is accelerated with the decrease of Al particle size.

**Fig. 7:**
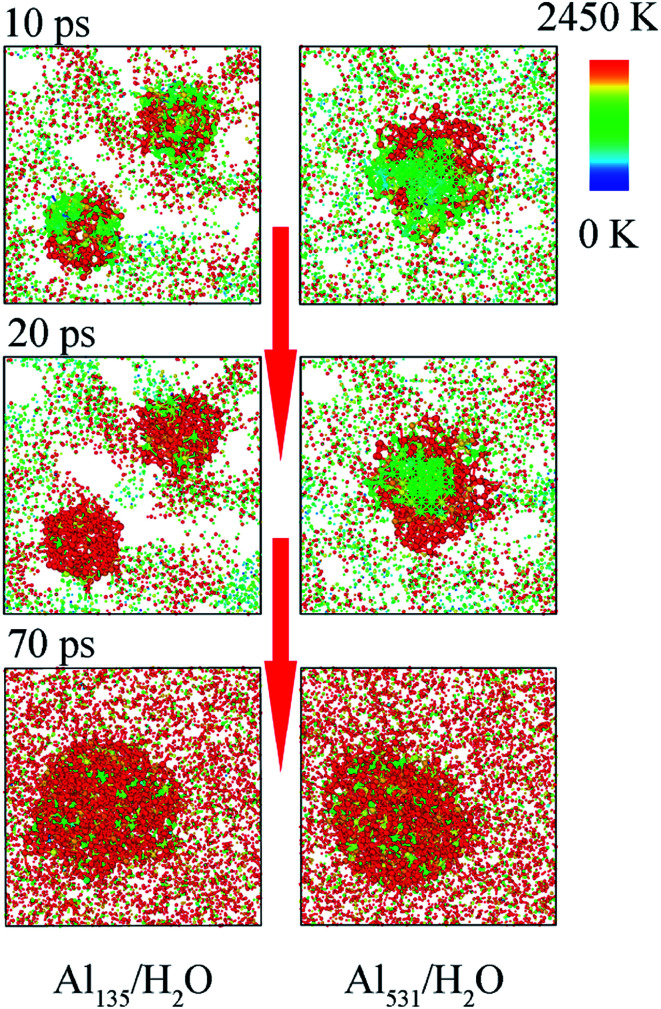
Sectional diagram of the temperature distribution.

Moreover, smaller particles are easier to heat, which can be supported by Fig. 7. The heating process of aluminum with different particle sizes is shown in Fig. 7. Both Al_135_ and Al_531_ begin to warm from the surface, corresponding to the snapshot at 10 ps. However, at 20 ps, Al_135_ has been completely heated, but Al_531_ is still only heated on the surface. Therefore, the faster overall participation of Al_135_ in the reaction with H_2_O is demonstrated. This is the third reason why the decrease of Al particle size accelerates the reaction rate. After 70 ps, four Al_135_ in the molten state showed agglomeration effect, forming an aluminum particle similar in size to Al_531_. Therefore, the difference in reaction rate only occurred between 20 and 70 ps.

As the aluminum particle size decreases, its specific surface area increases. At the same time, it is also more susceptible to heat and more easily to diffuse. These factors are the main reasons that particle size affects the reaction rate of aluminum with water. However, the agglomeration effect of aluminum particles in the later stage attenuates the effect of particle size on the reaction rate.

### 3.2 Reaction performance of surface passivated Al

**Fig. 8:**
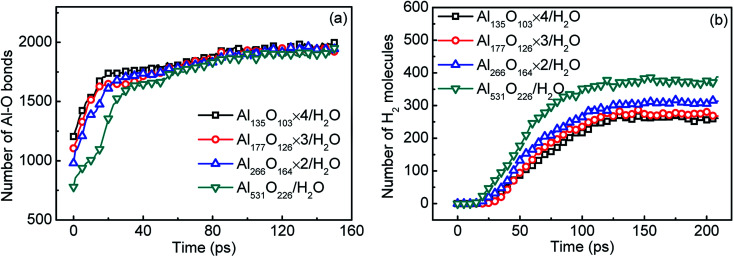
The number of Al–O bonds (a) and hydrogen yield (b) varied with time in systems containing surface passivated aluminum of different particle sizes.

The surface passivation of aluminum particles is inevitable in an oxygenated environment. The reaction performance of aluminum with water will be affected by the presence of passivated layer. In Fig. 8(a), the initial number of Al–O bonds increases with the decrease of Al particle size. This is because the specific surface area of aluminum increases as its particle size decreases. Therefore, the proportion of passivated part increases. Fig. 8(b) shows the relationship between the hydrogen yield and particle size. With the decrease of particle size, hydrogen yield decreases obviously. This is because the decrease in the proportion of active aluminum reduces the yield of hydrogen.

**Fig. 9:**
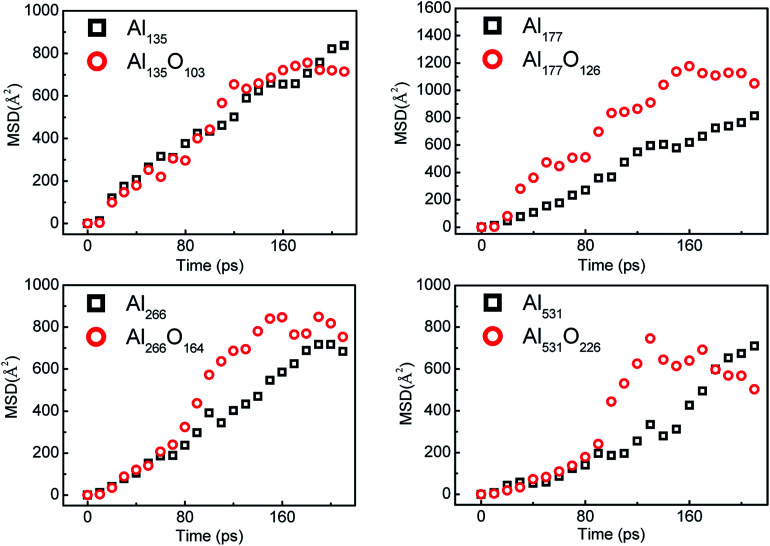
Mean-squared displacement (MSD) of Al atoms during the reaction of aluminum with water.

The mean-squared displacement (MSD) can reflect the motion of the atoms perfectly. During the reaction of aluminum with water, the MSD of aluminum atoms are shown in Fig. 9. MSD represents the square of the distance from the initial position of the atom, and it can reflect the degree of the atom's activity over time. Regardless of the size, the aluminum atoms in the surface passivated aluminum particles are more active than those in the pure aluminum particles in the middle stage of the reaction. This is because the aluminum atoms inside the particle react with alumina on the surface after the aluminum particle is passivated.

**Fig. 10:**
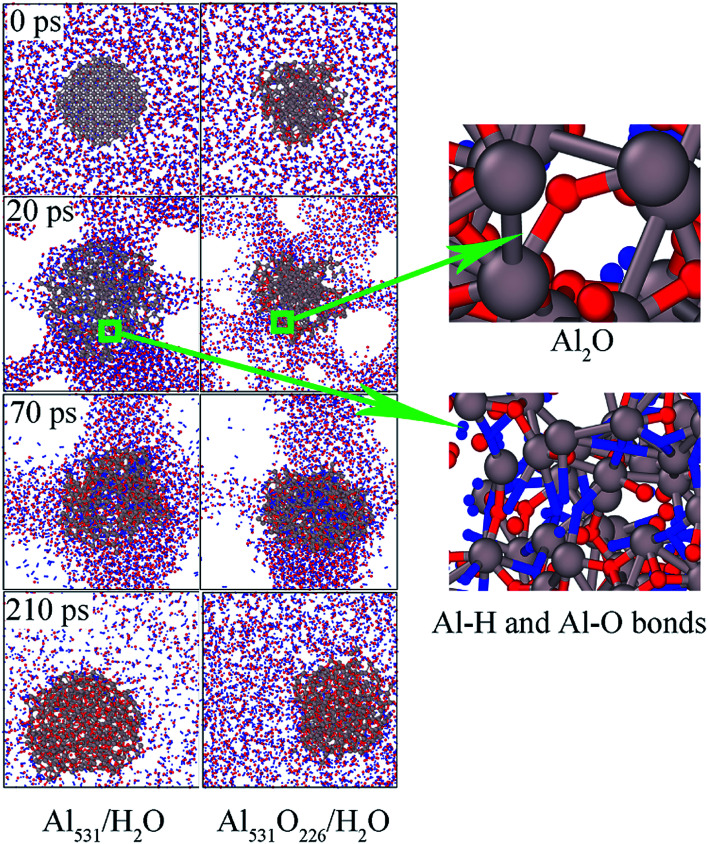
The snapshots of Al_531_/H_2_O and Al_531_O_226_/H_2_O over time. Al in gray, O in red and H in blue.

In order to explore the influence of surface passivation on the reaction mechanism, the snapshots of Al_531_/H_2_O and Al_531_O_226_/H_2_O systems as a function of time were presented in Fig. 10. When the reaction proceeds to 20 ps, the Al_531_ particle expands and generates Al–H and Al–O bonds from the surface, which corresponded to reaction (1) proposed in the previous study:^39^

1 3Al + 2H_2_O → 2AlO + AlH_3_ + H

However, Al_531_O_226_ does not expand, and there is no H atoms entering the interior due to the presence of surface oxidized layer.

At 70 ps, a large number of Al–H and Al–O bonds are formed inside the Al_531_ and Al_531_O_226_ due to the increase of temperature. But at the end of the reaction (210 ps), there is only alumina inside the residue, and H atoms have disappeared, proving that AlH_3_ is merely an intermediate in the reaction of Al with H_2_O.

**Fig. 11:**
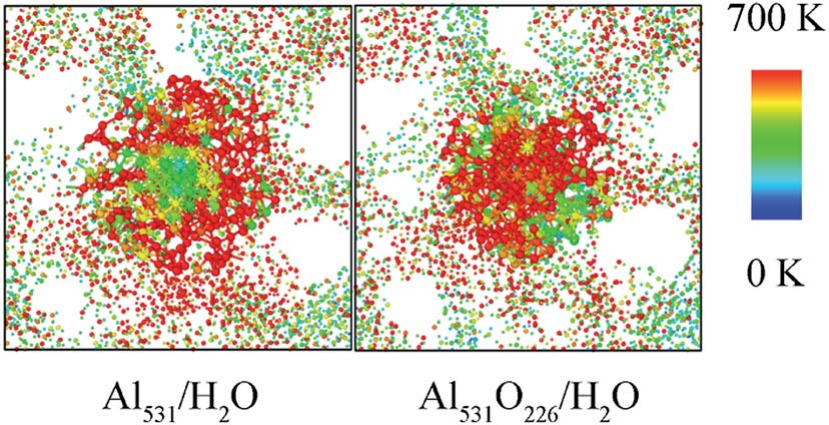
Temperature distributions of Al_531_/H_2_O and Al_531_O_226_/H_2_O systems at 20 ps.

In addition, the temperature distribution of Al_531_/H_2_O and Al_531_O_226_/H_2_O at 20 ps is shown in Fig. 11. Interestingly, the Al_531_ particle heats up from the surface, while the Al_531_O_226_ heats up from the inside. The Al atoms on the surface of Al_531_ react rapidly with H_2_O and release heat. However, no H atoms are observed in the interior of Al_531_O_226_ particles, proving that the interior Al atoms have not react with H_2_O at 20 ps. Combined with the snapshot in Fig. 10, the heat released is believed to come from the internal Al atoms reacting with oxidized layer:

2 4Al + Al_2_O_3_ → 3Al_2_O

Early experiments also reported the presence of Al_2_O in the combustion of surface passivated aluminum with water.^40^

Finally, the influence of the presence of the oxidized layer on the reaction performance with the change of particle size is statistically reported in the Table 4. With the decrease of aluminum particle size, both the proportion of active aluminum and the yield of hydrogen decrease. When the particle size of aluminum decreases from 2.5 nm to 1.6 nm, the content of active aluminum reduces by 30% and the yield of hydrogen even reduces by 33%.

**Table 4:** Effect of surface passivation on the reaction performance of aluminum and water with the particle size

Al in H_2_O	Active Al content (per cell)	Active Al reduction ratio (%)	H_2_ yield (per cell)	H_2_ yield reduction ratio (%)
Al_531_	531	0.0	530	0.0
Al_531_O_226_	380	28.4	388	26.8
Al_266_O_164_ × 2	313	41.1	314	40.7
Al_177_O_126_ × 3	279	47.5	268	49.4
Al_135_O_103_ × 4	265	50.9	261	50.8

### 3.3 Reactivity by adding AlH_3_ particle

**Fig. 12:**
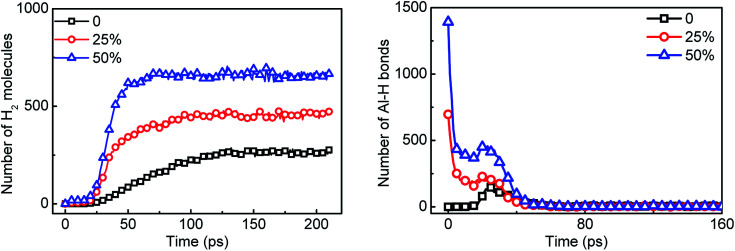
Statistics of hydrogen yield and the number of Al–H bonds in Al/AlH_3_/H_2_O system over time. 0, 25% and 50% are the replacement ratio of surface passivated Al by AlH_3_.

The presence of surface passivated layer greatly reduces the reaction performance of aluminum with water. In order to solve this problem, the surface passivated aluminum is replaced by AlH_3_ in proportion. In the Al_135_O_103_ × 4/H_2_O system, 25% and 50% of the surface passivated Al particles were replaced by AlH_3_, respectively. The number of H_2_ molecules and Al–H bonds are counted in the Fig. 12. As the replacement ratio increases, the hydrogen yield increases obviously. When the replacement ratio reaches 25%, the hydrogen yield is almost equal to that of the reaction between pure Al and H_2_O. Combined with the decrease in the amount of Al–H bonds, it is considered that AlH_3_ reacts with H_2_O through 2AlH_3_ + 3H_2_O → Al_2_O_3_ + 6H_2_ to release huge amounts of hydrogen.^39^ However, the number of AlH_3_ at about 30 ps increases abnormally since Al atoms react with H_2_O during this period as shown in Fig. 10 and generate AlH_3_ through 3Al + 2H_2_O → 2AlO + AlH_3_ + H.

**Fig. 13:**
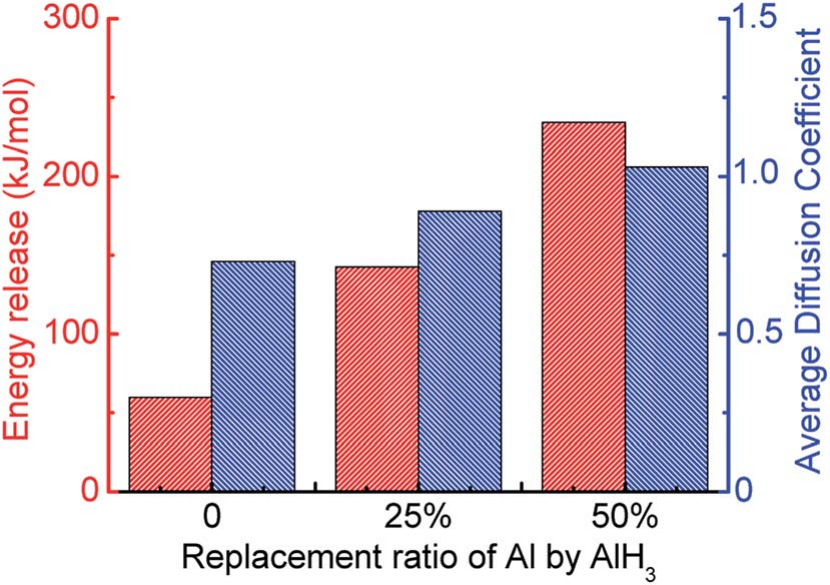
Energy released by Al/AlH_3_/H_2_O system and average diffusion coefficients of Al atoms.

Meanwhile, with the addition of AlH_3_, the generation rate of H_2_ is accelerated. Considering the increase in the diffusion coefficient of aluminum atoms in Fig. 13, it is believed that the addition of AlH_3_ increases the reactivity of the system. In addition, the energy release of the system is also shown in Fig. 13. When surface passivated aluminum particles with a diameter of 1.6 nm (Al_135_O_103_) react with water, only 59.47 kJ mol^−1^ of energy is released. The unit per mole here means per unit mixture contains one mole of aluminum. When 25% of the aluminum particles are replaced by AlH_3_, the system can release 142.56 kJ mol^−1^ of energy, which is close to that released by pure aluminum with water (180.67 kJ mol^−1^). When the molar ratio of AlH_3_ increases to 50%, this value goes up to 234.17 kJ mol^−1^.

## 4 Conclusion

The decomposition mechanism of H_2_O molecules on the surface of Al particles with different particle sizes was analyzed by using ReaxFF force field. With the decrease of the aluminum particle size, H_2_O molecules in the system consume faster in the medium term of the simulation. However, the increasing temperature in the later period will cause the agglomeration of Al particles to offset this effect.

Moreover, the influence of surface passivation of aluminum cannot be ignored as the particle size decreases. When the particle diameter decreases from 2.5 nm to 1.6 nm, the proportion of active aluminum decreases by 30%, and the hydrogen yield in the reaction with water decreases by 33% when the thickness of surface passivated shell is 0.35 nm. The addition of AlH_3_ can overcome this problem. When 25% of the surface passivated aluminum in the system is replaced by AlH_3_, the energy release increases from 59.47 kJ mol^−1^ to 142.56 kJ mol^−1^, and hydrogen yield increases form 0.0042 mol g^−1^ to 0.0076 g mol^−1^, which are close to those of pure Al/H_2_O system. To sum up, in the reaction of surface passivated aluminum with water, replacing a certain percentage of aluminum with AlH_3_ can offset the effect of passivated layer on hydrogen yield and energy content.

In addition, in the pure Al/H_2_O system, the heating starts at the interface between Al particles and H_2_O molecules, while in the surface passivated Al/H_2_O system, the heating starts inside the Al particle. This is because the reaction process 3Al + 2H_2_O → 2AlO + AlH_3_ + H occurs at the interface between pure Al and H_2_O. However, the passivated layer reacts with aluminum inside the particle through 4Al + Al_2_O_3_ → 3Al_2_O.

## Conflicts of interest

There are no conflicts to declare.

## Supplementary Material
